# Enhancing Exercise Responsiveness across Prediabetes Phenotypes by Targeting Insulin Sensitivity with Nutrition

**DOI:** 10.1155/2017/8314852

**Published:** 2017-12-13

**Authors:** Julian M. Gaitan, Arthur Weltman, Steven K. Malin

**Affiliations:** ^1^Department of Kinesiology, University of Virginia, Charlottesville, VA, USA; ^2^Division of Endocrinology and Metabolism, University of Virginia, Charlottesville, VA, USA; ^3^Robert M. Berne Cardiovascular Research Center, University of Virginia, Charlottesville, VA, USA

## Abstract

Exercise is a cornerstone therapy for chronic diseases related to multiorgan insulin resistance. However, not all individuals show the anticipated improvement in insulin sensitivity following exercise and these individuals are considered exercise resistant. Caloric restriction is an approach to enhance the effect of exercise on increasing peripheral and hepatic insulin sensitivity, as replenishing expended calories blunts these benefits. Alternatively, restricting carbohydrate intake, independent of energy balance, following exercise provides an additive effect on peripheral insulin sensitivity when compared to refeeding carbohydrate. Although carbohydrate composition modulates insulin sensitivity, few have studied effects of low glycemic index or whole-grain diets following exercise across prediabetes phenotypes on insulin sensitivity. Herein, we propose the novel hypothesis that the combination of individualized nutrition therapy and exercise should be based on the clinical pathology of prediabetes to overcome exercise resistance and improve responsiveness in people at risk for type 2 diabetes and cardiovascular disease.

## 1. Introduction

Approximately 382 million adults worldwide have type 2 diabetes (T2D), and this figure is projected to rise to 592 million by the year 2035 [1]. Prediabetes is characterized by blood glucose levels above normal but below the diagnostic criteria for T2D [2]. The etiology of prediabetes is primarily skeletal muscle, liver, and/or adipose tissue insulin resistance that, in time, promotes oversecretion of insulin from the *β*-cell and results in pancreatic exhaustion that leads to severe hyperglycemia [3].

Skeletal muscle insulin resistance is primarily responsible for impaired glucose tolerance (IGT; 2 hr postprandial glucose > 140 mg/dL). In contrast, hepatic insulin resistance manifests mainly as impaired fasting glucose (IFG; fasting plasma glucose > 100 mg/dL) because glucose production is excessive despite normal insulinemia [4]. Individuals with both IFG and IGT (i.e., IFG + IGT) have peripheral and hepatic insulin resistance. Obesity is considered a chief factor promoting this insulin resistance, and many individuals with IFG and/or IGT are advised to lose approximately 5–10% of body weight by consuming a low-fat diet and participating in at least 150 min/week of moderate or 75 min/week of vigorous intensity exercise [5]. However, there is substantial intersubject variability for glycemic control [6, 7], cardiometabolic health [8, 9], and skeletal muscle hypertrophy [10] in response to lifestyle intervention, despite exercise intensity influencing these fitness-related insulin sensitivity adaptations [11]. Those who respond with little improvement, or even with adverse response, are often termed “nonresponders” or “exercise resistant.” With regard to prediabetes, individuals with IFG + IGT have attenuated insulin sensitivity responses to high-volume (300 min/wk)/high-intensity (85% HR_max_) exercise with approximately 7% weight loss treatment as compared to IFG or IGT alone [6, 7]. This phenomenon represents a specific case of intersubject variability in response to a high dose of exercise. Interestingly, ad libitum changes in diet behavior were recently suggested to have tissue-specific effects on insulin sensitivity [12]. As IFG generally develops due to hepatic insulin resistance and IGT emerges from skeletal muscle insulin resistance, we propose the novel hypothesis that applying individualized dietary prescription may enable the insulin-sensitizing effects of exercise in exercise-resistant individuals based on the underlying hyperglycemic phenotype [6, 7, 9].

This review will examine the potential for diet to augment the benefits of exercise on glucose regulation in people with insulin resistance in the liver and skeletal muscle. In particular, attention will focus on the impact of energy balance, carbohydrate balance, and carbohydrate type (i.e., low glycemic index (GI) and whole grain) in conjunction with exercise on multiorgan insulin resistance and glycemic control. Acute exercise interventions consist of 1–10 days to reflect metabolic responsiveness, while chronic (~>8 weeks exposure) exercise studies are used to understand adaptations to the stimuli. These exercise conditions are discussed separately because insulin sensitivity effects may differ based on the length of exposure.

## 2. Energy Deficit

### 2.1. Acute Exercise

Energy deficit following acute exercise contributes to increased postexercise insulin sensitivity. In fact, an energy deficit of 6.5 kcal/kg body weight following an acute exercise bout resulted in a 22% lower postprandial insulin incremental area under the curve (AUC) during an oral glucose tolerance test (OGTT) compared with an 11% decrease when expended calories were replenished [13]. This suggests that approximately 50% of the exercise effect on insulin sensitivity is due to the exercise-induced energy deficit. Although use of an OGTT limits the ability to discern whether skeletal muscle or liver is responsible for the improvement, earlier work using clamp or stable isotope approaches confirm that energy deficit is at least partially responsible for increased insulin sensitivity. Assali et al. prescribed hypocaloric diets in two groups of obese individuals by caloric restriction of 33 or 66% of the habitual energy intake [14]. Peripheral insulin sensitivity as measured by the euglycemic clamp increased significantly in the first week of the intervention, before any documented change in weight. In 2 subsequent weeks when subjects were prescribed an isoenergetic diet, the same improvements in insulin sensitivity were lost. Consistent with these observations, Black et al. studied the effects of an energy deficit (500 kcal/day) induced by 6 days of exercise training on isotope-derived insulin sensitivity in overweight and obese adults in comparison to a group who had calories replenished following exercise [15]. After training, the energy deficit intervention increased peripheral glucose disposal and suppressed hepatic glucose production compared to energy repletion. In another acute study, a 10-day energy deficit via either moderate aerobic exercise training or a low-calorie diet (50% of kcal needed to maintain energy balance) in obese, IGT, and T2D subjects significantly increased insulin action as determined by a modified hyperglycemic clamp [16]. Despite similar energy deficits, exercise training had a significantly greater effect on improving insulin action than the low-calorie diet. Together, these studies support that energy deficit is an important mechanism accounting for increased skeletal muscle and hepatic insulin sensitivity, but that exercise training confers additional improvement. Thus, combining exercise with diet-induced energy deficit could be a manner of alleviating exercise resistance in groups such as IFG + IGT, but this remains to be tested.

### 2.2. Exercise Training

Long-term studies ranging from 6–12 months report that exercise-induced energy deficit is responsible for increasing insulin sensitivity. Weiss et al. demonstrated that energy deficit via caloric restriction or exercise after 12 months improved insulin sensitivity comparably [17]. This is consistent with work by Larson-Meyer et al. showing that when energy deficit was held constant through 25% caloric restriction or 12.5% caloric restriction plus 12.5% energy expenditure from aerobic exercise, there was no difference between groups in skeletal muscle insulin sensitivity improvements [18]. Moreover, in obese, nondiabetic African American women who underwent 14 weeks of high-intensity interval training (HIIT) with a weight-maintenance diet [19], there was no significant increase in insulin sensitivity when calories were refed. Collectively these studies suggest that energy deficit is important for explaining the improved insulin sensitivity seen with chronic exercise training. Indeed, when lean, obese, and T2D men underwent 12 weeks of aerobic exercise training and were refed sufficient calories (55% carbohydrate, 21% fat, and 24% protein) to maintain body weight, no group experienced improvements in peripheral insulin sensitivity as measured by the clamp, despite increased oxidative glucose disposal [20]. Nonetheless, basal hepatic glucose production was reduced in subjects with T2D following exercise plus diet, indicating an independent effect of exercise on liver glucose metabolism in people with T2D. More recent work suggests that an energy deficit of about 700 kcal/day by either restricting caloric intake (diet-induced weight loss) or 60 min/day of moderate intensity aerobic exercise (exercise-induced weight loss) resulted in similar increases in glucose tolerance and nonoxidative glucose disposal [21]. These findings are similar to those of Solomon et al. [22], who compared a eucaloric diet plus exercise (i.e., energy deficit by exercise) or a hypocaloric diet plus exercise (i.e., diet-imposed 500 kcal/day deficit plus exercise-induced energy deficit) in older, obese, sedentary adults. Individuals in the hypocaloric diet group lost approximately 4 kg more weight compared with people in the eucaloric group. Clamp-derived peripheral insulin sensitivity improved similarly in both groups following the 12 weeks of aerobic exercise training regardless of eucaloric or hypocaloric diet, suggesting that above a given threshold of weight loss, there may be no further improvement in insulin sensitivity. However, subsequent work by the same group showed that hepatic insulin sensitivity improved to a greater extent following exercise training with hypocaloric diet than with a eucaloric diet during lipid-infused conditions [23]. This suggests that in addition to exercise, calorie restriction may protect the liver from obesity-driven insulin resistance more so than training alone, despite comparable peripheral insulin sensitivity. These findings may suggest that people with hepatic insulin resistance derive more benefit by focusing on energy deficit than those people with IGT alone [23].

A confounding factor when interpreting the collective findings from the effects of acute and long-term energy deficit on insulin sensitivity is that a carbohydrate deficit is often induced concurrently. This raises the possibility that carbohydrate deficit (e.g., low-glycogen concentrations), and not energy deficit per se, regulates insulin sensitivity postexercise (see Carbohydrate Availability). Further work awaits investigation to elucidate the optimal nutrition program for maximizing improvements in hepatic and/or skeletal muscle insulin resistance during exercise interventions across the prediabetes phenotypes.

## 3. Carbohydrate Availability

### 3.1. Acute Exercise

Skeletal muscle glycogen concentration is a driver of insulin-stimulated glucose uptake [24], and exercise and dietary carbohydrate intake both affect muscle glycogen. In healthy young men, exercise-induced glycogen depletion combined with dietary carbohydrate restriction promotes insulin sensitivity [25], but no studies have tested this effect in subjects with hyperglycemia. Nonetheless, Holtz et al. [26] studied overweight, sedentary, normoglycemic adults and found that carbohydrate restriction after moderate exercise increased nonoxidative glucose disposal during glucose infusion to a greater extent than carbohydrate replenishment. This higher degree of carbohydrate restriction was strongly correlated with enhanced insulin action, suggesting that exercise-mediated glycogen depletion modulates the insulin-stimulated effect on skeletal muscle glucose uptake. In addition to the degree of carbohydrate restriction, the timing of nutrient intake may affect peripheral insulin sensitivity. After an exercise session, insulin action improved to the greatest extent when subjects were refed the expended calories and carbohydrate immediately after the exercise bout, as opposed to immediately before or 3 hr after exercise [27]. However, insulin-mediated hepatic glucose production was significantly suppressed with preexercise or 3 hrs delayed feeding compared with immediately postexercise feeding. Although these findings may not generalize to individuals with hyperglycemia, as the subjects tested were fit and of normal weight, the results suggest that the timing of energy and/or carbohydrate intake relative to exercise may alter whether peripheral or hepatic insulin sensitivity are most affected. If true in individuals with prediabetes, this would have implications for augmenting insulin sensitivity using exercise and nutrition prescriptions categorized by prediabetes phenotype.

To gain insight into the importance of carbohydrate restriction on enhancing exercise-induced insulin sensitivity, it is necessary to compare energy deficit and replenishment models that are matched for carbohydrate intake [25, 26, 28]. Studies on dietary energy deficit cannot distinguish whether exercise-induced insulin sensitivity is augmented by global calorie reduction or a specific effect of carbohydrate deficit. Those that compare postexercise dietary energy restriction plus carbohydrate balance, energy restriction plus carbohydrate restriction, and energy restriction plus carbohydrate balance are useful for understanding the importance of carbohydrate. They establish that carbohydrate is a key dietary variable for augmenting insulin sensitivity following exercise [29, 30]. Nevertheless, energy deficit even with carbohydrate replenishment enhances fat oxidation [29, 30], which may be advantageous for improving clamp and OGTT-derived insulin sensitivity during periods of energy deficit [31–33]. Similar to the study of Holtz et al. [26], greater improvement in insulin sensitivity coincided with greater reductions in skeletal muscle glycogen [30]. Given that chronic hyperglycemia attenuates skeletal muscle glycogen utilization during exercise [34–36], this observation of glycogen depletion could be important for individualizing exercise prescription if these results hold true in adults with T2D or IFG + IGT. Furthermore, this may explain to some extent why people with IFG + IGT appear exercise resistant and respond to exercise with smaller rises in insulin sensitivity [37]. Thus, adults with IFG + IGT could benefit from restricted postexercise carbohydrate intake of approximately 3 to 12 hrs [27, 30]. Further work is required to identify specific postexercise carbohydrate manipulations to increase insulin sensitivity among different prediabetes phenotypes.

Since insulin suppresses lipolysis via adipocyte insulin action, diets high in carbohydrate could reduce circulating lipids (barring high dietary fat intake) and diminish lipid-induced impairments on hepatic and/or skeletal muscle insulin sensitivity. This may be important for individuals with IGT, who tend to have more severe adipose insulin resistance than individuals with IFG [38]. Carbohydrate restriction after an exercise bout may enhance adipocyte insulin sensitivity. Harrison et al. [39] reported that circulating triglycerides (TG) were lower and free fatty acids (FFA) were higher during an oral fat tolerance test (OFTT) after an exercise bout and carbohydrate restriction. The effect as absent when carbohydrate was refed to replenish the exercise expenditure, suggesting adipocyte insulin sensitivity, was enhanced by dietary restriction after exercise. While muscle glycogen was substantially reduced by carbohydrate restriction, the study design makes it unclear whether carbohydrate deficit per se or energy deficit was responsible for reducing circulating TG. In either case, other work indicates that energy deficit decreases circulating TG in response to a high-fat meal thereby minimizing deleterious effects of lipids on insulin action [40]. To date, few data exist examining the impact of refeeding with or without carbohydrate on lipid availability as a result of insulin sensitivity. Further work is needed to understand the interaction among exercise, carbohydrate restriction, insulin sensitivity, and lipid availability, particularly in individuals with IGT who have adipose insulin resistance.

A final consideration with regard to restricting carbohydrate intake is that limiting one nutrient could be compensated by increased intake of another, such as fat. High dietary fat intake is thought to decrease insulin-stimulated skeletal muscle glucose uptake because of a rise in circulating lipids [41–44]. However, exercise may offer some protection. Several studies [45, 46] suggest that a 90 min aerobic exercise bout enhances peripheral and hepatic insulin sensitivity regardless of high- or low-fat feeding after the bout. Since skeletal muscle lipid oxidation capacity is correlated with insulin sensitivity in subjects with a high VO_2_peak and high IMTG content [47], these subjects' tolerance to high-fat meals may be partly explained by their aerobic fitness (VO_2_peak = 55.5 ± 1.6 mL/kg/min) [45] or the high oxidative capacity of females [46]. Further studies are needed to understand effects in clinical populations. Nonetheless, carbohydrate intake and muscle glycogen content were similar across conditions in these studies, thereby reinforcing the hypothesis that carbohydrate, and not fat, is a key dietary variable for enhancing exercise-induced insulin sensitivity in short term.

### 3.2. Exercise Training

While acute studies suggest that exercise with carbohydrate restriction may be effective for enhancing the insulin-sensitizing effect of exercise at the level of skeletal muscle and the liver, it is currently unknown whether this nutritional strategy is effective in conjunction with chronic exercise training in clinical populations. Subsequently, we can only speculate that since individuals with IFG + IGT tend to have blunted insulin sensitivity and fuel selection exercise responses to training [6, 7], carbohydrate restriction could be a strategy to enhance exercise-related mitochondrial adaptations in this population that relate to insulin sensitivity [48]. The mechanism by which low carbohydrate availability confers metabolic adaptation has been shown to act through targeting glycogen depletion to upregulate mitochondrial oxidative enzymes (e.g., citrate synthase, *β*-HAD) involved in glucose tolerance and insulin action [49, 50]. Further work is needed to determine whether exercise training with carbohydrate restriction is feasible in individuals across the prediabetes phenotypes.

## 4. Glycemic Index

### 4.1. Acute Exercise

The glycemic index (GI) of a food is characterized by the resulting blood glucose excursion with reference to a standardized meal (e.g., white bread or glucose) [51]. On the other hand, the glycemic load (GL) is determined by the GI of the food multiplied by the amount of carbohydrate (i.e., grams) in that serving, also described by the area under the curve of the GI response. It is worth acknowledging, however, that GI and GL are independent of dietary energy, which has been an important topic of this review. Nevertheless, the purpose of a low-GI or low-GL diet is to manage circulating glucose levels (and by association, insulin levels), which in turn, may contribute to healthier body weight, reduced appetite cravings, and improved cardiometabolic health [51, 52]. Although few studies have systematically compared the effect of low versus high GI food following an acute bout of exercise on insulin sensitivity, it should be noted that many of the acute exercise studies examined in this review utilized a high content of simple carbohydrates, which have a high GI index. In one of the few acute studies conducted, Solomon et al. [53] examined older, obese adults after 7 days of moderate intensity aerobic exercise together with a low- or high-GI diet. Exercise combined with low- and high-GI diets enhanced insulin-stimulated glucose disposal and hepatic glucose production (markers of peripheral and hepatic insulin sensitivity). Since these two diets improved insulin sensitivity in both tissues, further work is warranted to determine whether IFG, IGT, and IFG + IGT phenotypes respond differently to each diet. Although the low-GI diet did not improve insulin sensitivity more than high GI following acute exercise, the low-GI group reduced systolic blood pressure to a greater extent. Thus, a low-GI diet with exercise may contribute to short-term cardiovascular benefits, presenting a possible strategy to mitigate cardiovascular disease if exercise is performed regularly. This may be clinically important for the prediabetes phenotypes since individuals with IFG + IGT have higher CVD risk than those with IGT or IFG [54].

### 4.2. Exercise Training

Exercise training and a eucaloric low-GI diet have been suggested to synergistically enhance glucose regulation in older adults with prediabetes [55]. Despite comparable improvements in peripheral and hepatic insulin sensitivity, exercise training combined with a low-GI diet can preserve *β*-cell function and reduce insulin secretion demand to a greater extent than a high-GI diet [56]. Preserving *β*-cell function is relevant to all three prediabetes phenotypes (IFG, IGT, and IFG + IGT), since pancreatic dysfunction is differentially implicated in the progression to T2D [57]. On the other hand, Malin et al. [58] showed that 12 weeks of either a low- or high-GI diet and supervised exercise training was effective in lowering HbA_1c_ and body fat as well as increasing peripheral insulin sensitivity in adults with metabolic syndrome, without change in resting fat oxidation. Consistent with this observation, a low-GI food consumption combined with increased walking in adults with T2D resulted in reductions of HbA_1c_ that were similar to increased walking alone [59]. When drawing on these observations, there does not appear to be clear evidence that a eucaloric low-GI diet confers benefit above that of a high-GI diet when combined with increased physical activity and/or exercise training. Additional research is needed to understand differential responses in each prediabetic phenotype to better understand personalizing diets with exercise on insulin sensitivity and/or secretion.

## 5. Whole Grains

To date, there are no systematic acute or long-term exercise interventions combined with whole-grain consumption examining effects on insulin sensitivity in hyperglycemic individuals. However, epidemiological data suggest that dietary whole-grain intake (e.g., oats and barely) is associated with a lower incidence of T2D compared with refined-grain intake [60]. Whole-grain consumption (~30–60 g/d) may also reduce the risk of progression from normal glucose tolerance to prediabetes [61]. Indeed, Kirwan et al. [62] recently demonstrated that 8 weeks of whole-grain diet (consisting of mainly wheat and rice) feeding decreased HbA_1c_, fasting insulinemia, and insulin resistance (HOMA-IR) in overweight/obese subjects. The reason whole-grain intake lowers T2D risk is an area ready for investigation as preliminary work suggests that short-chain fatty acid release in the gut mediates the effect of whole-grain (bran and germ with high fiber) intake on clamp-derived insulin sensitivity in overweight adults with or without metabolic syndrome [63]. This suggests that whole-grain intake may improve skeletal muscle glucose uptake or reduce hepatic glucose production regardless of the metabolic derangement. However, whole grains have little effect on rates of meal glucose appearance from the gut in response to a standard OGTT with the dual-tracer technique [64, 65]. A single meal consisting of whole grains (barley-based meals rich in the soluble fiber *β*-glucan) results in better glycemic control than refined grains due to lower hepatic glucose production [65]. In addition, longitudinal data support that whole-grain consumption (rye bread based) may result in improved *β*-cell function [66]. It is worth recognizing though that dietary fiber is often associated with whole-grain intake and has shown independent effects on insulin sensitivity [67, 68]. Collectively, these studies support that whole-grain intake can improve peripheral and hepatic insulin sensitivity, along with pancreatic function. The results are thus relevant to IFG, IGT, and IFG + IGT populations, and particular attention should be paid to IFG + IGT since these individuals are most at risk for progression to T2D and may benefit from the combined peripheral and hepatic effects of whole-grain intake. However, whether adding whole grains and/or fiber augments, exercise effects above remain to be seen. This could be of clinical interest since exercise is a significant variable when studying the relationship between fiber intake and insulin resistance [67]. Future research is needed to examine responses in each obese prediabetic phenotype to the interaction between whole grains with acute and long-term exercise on insulin sensitivity for T2D prevention/treatment.

## 6. Conclusions

Modifying dietary energy and nutrient intake is an appropriate tool to enhance the effectiveness of exercise on insulin sensitivity. Various dietary strategies appear to have independent effects on targeting skeletal muscle versus hepatic insulin sensitivity (Table 1). For instance, inducing an energy deficit improves skeletal muscle and hepatic insulin sensitivity following acute and chronic exercise interventions, which has implications for individuals with IFG, IGT, and IFG + IGT. Carbohydrate restriction is primarily responsible for depleting and maintaining low skeletal muscle glycogen, which enhances peripheral glucose uptake further than energy deficit with carbohydrate balance. Individuals with IGT, and especially IFG + IGT (as this group is more resistant to insulin sensitizing effects of exercise [6, 7]), may benefit from restricting carbohydrate intake following exercise bouts. Increased fasting fat oxidation is also consistently observed after exercise with calorie and/or carbohydrate restriction, suggesting that enhanced mitochondrial reliance on fat may contribute to improved insulin sensitivity. Effects of low- versus high-GI carbohydrates are unclear, but the literature supports that exercise followed by high-GI carbohydrate intake increases pancreatic insulin secretion. The pancreas is thus potentially subjected to additional burden in order to maintain normal glycemia. This is undesirable in IFG, IGT, and IFG + IGT phenotypes as pancreatic *β*-cell dysfunction contributes in the progression to T2D [69]. Whether whole grains modulate exercise adaption also remains to be seen. This is important as nutritional interventions suggest that adherence to low or high carbohydrate-based diets overtime may be most important for weight loss and glycemic control [70, 71].

A substantial number of the studies on interaction of nutrition and exercise for insulin action have been conducted in nonclinical populations. Clinical trials are needed to determine the efficacy of diet plus exercise for T2D prevention, particularly across the prediabetes phenotypes. Although individuals are often advised to consume nutrients at specific times before and after exercise to stimulate physiological adaptation and maximize athletic performance [72–74], the implication of nutrient composition and timing for individuals with IFG, IGT, and IFG + IGT remains largely unknown. Furthermore, no data exist examining the interaction between exercise intensity and nutrient composition and timing. These are significant knowledge gaps to fill since the pathophysiology of prediabetes differs among the clinical phenotypes. Addressing these questions will eventually allow individualized diet and exercise prescription and inform clinical and public health recommendations for optimizing the prevention and/or delay of T2D onset.

## Figures and Tables

**Table 1:** Summary of exercise plus diet effects compared with preintervention.

	Insulin-stimulated muscle glucose uptake	Nonoxidative glucose disposal	Glycogen concentration	Hepatic insulin sensitivity	Fat oxidation
Caloric restriction	↑ [15, 22]	↑ [15]	↔ [30]	↓ [16]	↑ [22, 29, 30]
Caloric replacement	↑ ↔ [15, 20–22, 75]	↓↔ [15, 20]	↔ [30, 39, 46]	↑↔ [15, 20]	↑↔ [13, 19, 30]
CHO restriction	↑↑ [26, 30]	↑↑ [26]	↓ [30, 39]	↔ [26]	↑↔ [30]
CHO Replacement	↔ [30]	↑↔ [26, 27]	↔ [30]	↔ [27]	↑ [27]
Low GI	↑ [56]	↑ [52]	?	↑ [52]	?
High GI	↑ [52]	↑ [52]	?	↑ [52]	?
Whole grain	?	?	?	?	?
Refined grain	?	?	?	?	?

↑ indicates an increase. ↓ indicates a decrease. ↔ indicates no substantial change. ? indicates inconsistent findings or no available data across studies. ↑↑ indicates a substantial change.

## References

[1] Guariguata L., Whiting D. R., Hambleton I., Beagley J., Linnenkamp U., Shaw J. E. (2014). Global estimates of diabetes prevalence for 2013 and projections for 2035. *Diabetes Research and Clinical Practice*.

[2] American Diabetes Association (2015). 2. Classification and Diagnosis of Diabetes. *Diabetes Care*.

[3] Fonseca V. A. (2009). Defining and characterizing the progression of type 2 diabetes. *Diabetes Care*.

[4] Meyer C., Pimenta W., Woerle H. J. (2006). Different mechanisms for impaired fasting glucose and impaired postprandial glucose tolerance in humans. *Diabetes Care*.

[5] Knowler W. C., Barrett-Connor E., Fowler S. E. (2002). Reduction in the incidence of type 2 diabetes with lifestyle intervention or metformin. *The New England Journal of Medicine*.

[6] Malin S. K., Haus J. M., Solomon T. P. J., Blaszczak A., Kashyap S. R., Kirwan J. P. (2013). Insulin sensitivity and metabolic flexibility following exercise training among different obese insulin-resistant phenotypes. *American Journal of Physiology - Endocrinology and Metabolism*.

[7] Solomon T. P. J., Malin S. K., Karstoft K., Haus J. M., Kirwan J. P. (2013). The influence of hyperglycemia on the therapeutic effect of exercise on glycemic control in patients with type 2 diabetes mellitus. *JAMA Internal Medicine*.

[8] Bouchard C., Blair S. N., Church T. S. (2012). Adverse metabolic response to regular exercise: is it a rare or common occurrence?. *PLoS One*.

[9] Green D. J., Eijsvogels T., Bouts Y. M. (2014). Exercise training and artery function in humans: nonresponse and its relationship to cardiovascular risk factors. *Journal of Applied Physiology*.

[10] Thalacker-Mercer A., Stec M., Cui X., Cross J., Windham S., Bamman M. (2013). Cluster analysis reveals differential transcript profiles associated with resistance training-induced human skeletal muscle hypertrophy. *Physiological Genomics*.

[11] Alvarez C., Ramirez-Campillo R., Ramirez-Velez R., Izquierdo M. (2017). Prevelance of non-responders for glucose control markers after 10 weeks of high intensity interval training in adult women with higher and lower insulin resistance. *Frontiers in Physiology*.

[12] Blanco-Rojo R., Alcala-Diaz J. F., Wopereis S. (2016). The insulin resistance phenotype (muscle or liver) interacts with the type of diet to determine changes in disposition index after 2 years of intervention: the CORDIOPREV-DIAB randomised clinical trial. *Diabetologia*.

[13] Burton F. L., Malkova D., Caslake M. J., Gill J. M. R. (2008). Energy replacement attenuates the effects of prior moderate exercise on postprandial metabolism in overweight/obese men. *International Journal of Obesity*.

[14] Assali A. R., Ganor A., Beigel Y., Shafer Z., Hershcovici T., Fainaru M. (2001). Insulin resistance in obesity: body-weight or energy balance?. *The Journal of Endocrinology*.

[15] Black S. E., Mitchell E., Freedson P. S., Chipkin S. R., Braun B. (2005). Improved insulin action following short-term exercise training: role of energy and carbohydrate balance. *Journal of Applied Physiology*.

[16] Arciero P. J., Vukovich M. D., Holloszy J. O., Racette S. B., Kohrt W. M. (1999). Comparison of short-term diet and exercise on insulin action in individuals with abnormal glucose tolerance. *Journal of Applied Physiology*.

[17] Weiss E. P., Racette S. B., Villareal D. T. (2006). Improvements in glucose tolerance and insulin action induced by increasing energy expenditure or decreasing energy intake: a randomized controlled trial. *The American Journal of Clinical Nutrition*.

[18] Larson-Meyer D. E., Heilbronn L. K., Redman L. M. (2006). Effect of calorie restriction with or without exercise on insulin sensitivity, *β*-cell function, fat cell size, and ectopic lipid in overweight subjects. *Diabetes Care*.

[19] Arad A. D., DiMenna F. J., Thomas N. (2015). High-intensity interval training without weight loss improves exercise but not basal or insulin-induced metabolism in overweight/obese African American women. *Journal of Applied Physiology*.

[20] Segal K. R., Edano A., Abalos A. (1991). Effect of exercise training on insulin sensitivity and glucose metabolism in lean, obese, and diabetic men. *Journal of Applied Physiology*.

[21] Ross R., Dagnone D., Jones P. J. (2000). Reduction in obesity and related comorbid conditions after diet-induced weight loss or exercise-induced weight loss in men: a randomized, controlled trial. *Annals of Internal Medicine*.

[22] Solomon T. P. J., Sistrun S. N., Krishnan R. K. (2008). Exercise and diet enhance fat oxidation and reduce insulin resistance in older obese adults. *Journal of Applied Physiology*.

[23] Haus J. M., Solomon T. P., Marchetti C. M., Edmison J. M., Gonzalez F., Kirwan J. P. (2010). Free fatty acid-induced hepatic insulin resistance is attenuated following lifestyle intervention in obese individuals with impaired glucose tolerance. *The Journal of Clinical Endocrinology & Metabolism*.

[24] Wojtaszewski J. F. P., Nielsen J. N., Richter E. A. (2002). Invited review: effect of acute exercise on insulin signaling and action in humans. *Journal of Applied Physiology*.

[25] Sparti A., Decombaz J. (1992). Effect of diet on glucose tolerance 36 hours after glycogen-depleting exercise. *European Journal of Clinical Nutrition*.

[26] Holtz K. A., Stephens B. R., Sharoff C. G., Chipkin S. R., Braun B. (2008). The effect of carbohydrate availability following exercise on whole-body insulin action. *Applied Physiology, Nutrition, and Metabolism*.

[27] Stephens B. R., Sautter J. M., Holtz K. A., Sharoff C. G., Chipkin S. R., Braun B. (2007). Effect of timing of energy and carbohydrate replacement on post-exercise insulin action. *Applied Physiology, Nutrition, and Metabolism*.

[28] Cartee G. D., Young D. A., Sleeper M. D., Zierath J., Wallberg-Henriksson H., Holloszy J. O. (1989). Prolonged increase in insulin-stimulated glucose transport in muscle after exercise. *American Journal of Physiology - Endocrinology and Metabolism*.

[29] Horowitz J. F., Kaufman A. E., Fox A. K., Harber M. P. (2005). Energy deficit without reducing dietary carbohydrate alters resting carbohydrate oxidation and fatty acid availability. *Journal of Applied Physiology*.

[30] Newsom S. A., Schenk S., Thomas K. M. (2010). Energy deficit after exercise augments lipid mobilization but does not contribute to the exercise-induced increase in insulin sensitivity. *Journal of Applied Physiology*.

[31] Goodpaster B. H., Katsariaras A., Kelley D. E. (2003). Enhanced fat oxidation through physical activity is associated with improvements in insulin sensitivity in obesity. *Diabetes*.

[32] Lattuada G., Costantino F., Caumo A. (2005). Reduced whole-body lipid oxidation is associated with insulin resistance, but not with intramyocellular lipid content in offspring of type 2 diabetic patients. *Diabetologia*.

[33] Venables M. C., Jeukendrup A. E. (2008). Endurance training and obesity: effect on substrate metabolism and insulin sensitivity. *Medicine and Science in Sports and Exercise*.

[34] Braun B., Sharoff C., Chipkin S. R., Beaudoin F. (2004). Effects of insulin resistance on substrate utilization during exercise in overweight women. *Journal of Applied Physiology*.

[35] Kang J., Kelley D. E., Robertson R. J. (1999). Substrate utilization and glucose turnover during exercise of varying intensities in individuals with NIDDM. *Medicine & Science in Sports & Exercise*.

[36] Malin S. K., Viskochil R., Oliver C., Braun B. (2013). Mild fasting hyperglycemia shifts fuel reliance toward fat during exercise in adults with impaired glucose tolerance. *Journal of Applied Physiology*.

[37] Malin S. K., Solomon T. P. J., Blaszczak A., Finnegan S., Filion J., Kirwan J. P. (2013). Pancreatic *β*-cell function increases in a linear dose-response manner following exercise training in adults with prediabetes. *American Journal of Physiology - Endocrinology and Metabolism*.

[38] Abdul-Ghani M. A., Molina-Carrion M., Jani R., Jenkinson C., DeFronzo R. A. (2008). Adipocytes in subjects with impaired fasting glucose and impaired glucose tolerance are resistant to the anti-lipolytic effect of insulin. *Acta Diabetologica*.

[39] Harrison M., O’Gorman D. J., McCaffrey N. (2009). Influence of acute exercise with and without carbohydrate replacement on postprandial lipid metabolism. *Journal of Applied Physiology*.

[40] Freese E. C., Levine A. S., Chapman D. P., Hausman D. B., Cureton K. J. (2011). Effects of acute sprint interval cycling and energy replacement on postprandial lipemia. *Journal of Applied Physiology*.

[41] Boden G. (1997). Role of fatty acids in the pathogenesis of insulin resistance and NIDDM. *Diabetes*.

[42] Boden G., Chen X. (1995). Effects of fat on glucose uptake and utilization in patients with non-insulin-dependent diabetes. *The Journal of Clinical Investigation*.

[43] Boden G., Chen X., Ruiz J., White J. V., Rossetti L. (1994). Mechanisms of fatty acid-induced inhibition of glucose uptake. *The Journal of Clinical Investigation*.

[44] Yu C. (2002). Mechanism by which fatty acids inhibit insulin activation of insulin receptor substrate-1 (IRS-1)-associated phosphatidylinositol 3-kinase activity in muscle. *The Journal of Biological Chemistry*.

[45] Fox A. K., Kaufman A. E., Horowitz J. F. (2004). Adding fat calories to meals after exercise does not alter glucose tolerance. *Journal of Applied Physiology*.

[46] Schenk S., Cook J. N., Kaufman A. E., Horowitz J. F. (2004). Postexercise insulin sensitivity is not impaired after an overnight lipid infusion. *American Journal of Physiology - Endocrinology and Metabolism*.

[47] Goodpaster B. H., He J., Watkins S., Kelley D. E. (2001). Skeletal muscle lipid content and insulin resistance: evidence for a paradox in endurance-trained athletes. *The Journal of Clinical Endocrinology and Metabolism*.

[48] Kelley D. E., Mandarino L. J. (2000). Fuel selection in human skeletal muscle in insulin resistance: a reexamination. *Diabetes*.

[49] Van Proeyen K., Szlufcik K., Nielens H. (2010). Training in the fasted state improves glucose tolerance during fat-rich diet. *The Journal of Physiology*.

[50] Jeukendrup A. E. (2017). Periodized nutrition for athletes. *Sports Medicine*.

[51] Evert A. B., Boucher J. L., Cypress M. (2014). Nutrition therapy recommendations for the management of adults with diabetes. *Diabetes Care*.

[52] Visuthranukul C., Sirimongkol P., Prachansuwan A., Pruksananonda C., Chomtho S. (2015). Low-glycemic index diet may improve insulin sensitivity in obese children. *Pediatric Research*.

[53] Solomon T. P., Haus J. M., Kelly K. R. (2009). Randomized trial on the effects of a 7-d low-glycemic diet and exercise intervention on insulin resistance in older obese humans. *The American Journal of Clinical Nutrition*.

[54] Abdul-Ghani M., DeFronzo R. A., Amin J. (2016). Prediabetes and risk of diabetes and associated complications. *Current Opinion in Clinical Nutrition and Metabolic Care*.

[55] Kirwan J. P., Barkoukis H., Brooks L. M., Marchetti C. M., Stetzer B. P., Gonzalez F. (2009). Exercise training and dietary glycemic load may have synergistic effects on insulin resistance in older obese adults. *Annals of Nutrition & Metabolism*.

[56] Solomon T. P., Haus J. M., Kelly K. R. (2010). A low-glycemic index diet combined with exercise reduces insulin resistance, postprandial hyperinsulinemia, and glucose-dependent insulinotropic polypeptide responses in obese, prediabetic humans. *The American Journal of Clinical Nutrition*.

[57] Færch K., Borch-Johnsen K., Holst J. J., Vaag A. (2009). Pathophysiology and aetiology of impaired fasting glycaemia and impaired glucose tolerance: does it matter for prevention and treatment of type 2 diabetes?. *Diabetologia*.

[58] Malin S. K., Niemi N., Solomon T. P. J. (2012). Exercise training with weight loss and either a high- or low-glycemic index diet reduces metabolic syndrome severity in older adults. *Annals of Nutrition & Metabolism*.

[59] Cheong S. H., McCargar L. J., Paty B. W., Tudor-Locke C., Bell R. C. (2009). The first step first bite program: guidance to increase physical activity and daily intake of low–glycemic index foods. *Journal of the American Dietetic Association*.

[60] Ye E. Q., Chacko S. A., Chou E. L., Kugizaki M., Liu S. (2012). Greater whole-grain intake is associated with lower risk of type 2 diabetes, cardiovascular disease, and weight gain. *The Journal of Nutrition*.

[61] Wirstrom T., Hilding A., HF G., Ostenson C.-G., Bjorklund A. (2013). Consumption of whole grain reduces risk of deteriorating glucose tolerance, including progression to prediabetes. *The American Journal of Clinical Nutrition*.

[62] Kirwan J. P., Malin S. K., Scelsi A. R. (2016). A whole-grain diet reduces cardiovascular risk factors in overweight and obese adults: a randomized controlled trial. *The Journal of Nutrition*.

[63] Pereira M. A., Jacobs D. R., Pins J. J. (2002). Effect of whole grains on insulin sensitivity in overweight hyperinsulinemic adults. *The American Journal of Clinical Nutrition*.

[64] Priebe M. G., Wang H., Weening D., Schepers M., Preston T., Vonk R. J. (2010). Factors related to colonic fermentation of nondigestible carbohydrates of a previous evening meal increase tissue glucose uptake and moderate glucose-associated inflammation. *The American Journal of Clinical Nutrition*.

[65] Thorburn A., Muir J., Proietto J. (1993). Carbohydrate fermentation decreases hepatic glucose output in healthy subjects. *Metabolism*.

[66] Juntunen K. S., Laaksonen D. E., Poutanen K. S., Niskanen L. K., Mykkänen H. M. (2003). High-fiber rye bread and insulin secretion and sensitivity in healthy postmenopausal women. *The American Journal of Clinical Nutrition*.

[67] Breneman C. B., Tucker L. (2013). Dietary fibre consumption and insulin resistance – the role of body fat and physical activity. *The British Journal of Nutrition*.

[68] Heikkilä H. M., Krachler B., Rauramaa R., Schwab U. S. (2014). Diet, insulin secretion and insulin sensitivity – the dose–responses to exercise training (DR’s EXTRA) study (ISRCTN45977199). *The British Journal of Nutrition*.

[69] Kanat M., Winnier D., Norton L. (2011). The relationship between β-cell function and glycated hemoglobin: results from the veterans administration epidemiology study. *Diabetes Care*.

[70] Rock C. L., Flatt S. W., Pakiz B. (2014). Weight loss, glycemic control, and cardiovascular disease risk factors in response to differential diet composition in a weight loss program in type 2 diabetes: a randomized controlled trial. *Diabetes Care*.

[71] Sacks F. M., Carey V. J., Anderson C. A. M. (2014). Effects of high vs low glycemic index of dietary carbohydrate on cardiovascular disease risk factors and insulin sensitivity. *Journal of the American Medical Association*.

[72] Ivy J. L., Lee M. C., Brozinick J. T., Reed M. J. (1988). Muscle glycogen storage after different amounts of carbohydrate ingestion. *Journal of Applied Physiology*.

[73] Jentjens R., Jeukendrup A. E. (2003). Determinants of post-exercise glycogen synthesis during short-term recovery. *Sports Medicine*.

[74] MacDougall J. D., Ward G. R., Sutton J. R. (1977). Muscle glycogen repletion after high-intensity intermittent exercise. *Journal of Applied Physiology*.

[75] Ross R., Janssen I., Dawson J. (2004). Exercise-induced reduction in obesity and insulin resistance in women: a randomized controlled trial. *Obesity Research*.

